# High Resolution Images of Human Meibum Spread on Saline

**DOI:** 10.1167/iovs.65.8.41

**Published:** 2024-07-24

**Authors:** P. Ewen King-Smith, Carolyn G. Begley, Richard J. Braun

**Affiliations:** 1 College of Optometry, Ohio State University, Columbus, Ohio, United States; 2 School of Optometry, Indiana University, Bloomington, Indiana, United States; 3 Department of Mathematical Sciences, University of Delaware, Newark, Delaware, United States

**Keywords:** meibum, tear film, evaporative dry eye

## Abstract

**Purpose:**

Understanding of the role of the tear film lipid layer (TFLL) in evaporative dry eye requires knowledge of its structure. X-ray studies show 11.1-nm thick lamellae in meibum at tear film temperature (approximately 35°C), whereas below 30°C, 4.88-nm thick lamellae predominate. Here, high resolution microscopy of meibum spread on saline is studied as a function of temperature, to compare with x-ray results.

**Methods:**

A purpose-built high resolution color microscope, previously used to study the TFLL, was used to study meibum from 10 subjects. It was spread on buffered saline at near 40°C, and allowed to cool to room temperature. Analytical methods from previous studies were applied to measure meibum and lamellar thickness.

**Results:**

Initially, an irregular “island” was formed, surrounded by a “background layer” of 7.8 ± 0.3 nm thickness. Dewetting of the meibum layer always occurred, leading to the formation of lens-shaped droplets. Below 30°C, the lenses start to emit “tails” having a multilamellar structure containing up to about 49 lamellae superimposed on the background layer, each lamella being 4.82 ± 0.13 nm thick.

**Conclusions:**

Below 30°C, meibum spread on saline shows a multilamellar structure like the 4.88 nm thickness in x-ray studies, demonstrating the ability to observe and measure tightly stacked lamellae. In contrast, above 30°C, the 11.1 nm lamellae were not observed as in x-ray studies, indicating that these lamellae were not tightly stacked but may be separated by disordered lipid. The role of these findings in evaporative dry eye is discussed.

Modeling of the tear film lipid layer (TFLL) should include consideration of evidence for lamellar structures in meibum. The lipid droplets in mouse meibomian glands exhibit a multilayered lamellar structure in transmission electron micrographs. The lamellae consist of alternating dark and light layers of 4 to 5 nm thickness, resembling liquid crystals and implying a lamellar spacing (periodicity) of 8 to 10 nm.^1^ A similar “onion-like” multilamellar structure of human lipid droplets can be shown by freeze fracture electron microscopy.^2^ Small angle x-ray scattering (SAXS) of human bulk meibum shows a multilayered structure with lamellar spacing of 4.88 nm that is the dominant structure below 30°C, but these lamellae melt at 34°C. Above 30°C, a structure with lamellar spacing of 11.1 nm increases in amplitude, and so becomes the main structure at an eye temperature of approximately 35°C.^3^ By analogy to the lipid layers in the skin, where 2 different lamellar spacings are also found,^4^ the 4.88 nm spacing will be called the short period phase (SPP) and the 11.1 nm spacing the long period phase (LPP).

Two models of the TFLL have included information from these SAXS results. In one “crystallite” model, the lamellar structures are thought to be small crystallites embedded in a viscoelastic, fluid continuous phase; this suspension is superimposed on a layer of polar lipids, forming the interface with the muco-aqueous layer. The crystallites are thought to impart strong viscoelasticity to the TFLL.^5^ Because the crystallites are so small – less than the thickness of the lipid layer – they would have little effect on reducing evaporation through the surrounding fluid lipid.

An alternative “evaporation” model was proposed based on evidence that the TFLL is a barrier to evaporation,^6^ like the skin. The skin lipid layer contains tightly stacked lamellae.^4^ It was therefore proposed that the TFLL has a similar structure of continuous, tightly stacked, nonpolar lamellae, superimposed on a thin, polar lamella.^7^ The nonpolar lamellae were thought to contain interdigitated, long saturated chains, for example, cholesteryl esters toward one surface and wax esters toward the other, as in Figure 4E of that paper.^7^ The dense array of long saturated chains would be expected to present a good barrier to evaporation.^8^

This study observed the effect of temperature on images of meibum spread on saline, to compare with SAXS results^3^ and with expectations from the crystallite and evaporative models. Optical imaging of meibum on saline is typically performed with Brewster angle microscopy (BAM).^3^^,^^9^ An advantage of BAM is that it can be used to detect and measure very thin lipid layers, for example, 1 nm thick.^10^ However, for this study, we preferred to use reflection, high resolution color microscopy (HRCM) for three reasons. First, HRCM has higher lateral resolution, about 1 µm^11^ compared to about 10 µm for BAM.^3^ Second, because BAM typically uses a monochrome camera and reflectance is an oscillatory function of lipid thickness,^11^ a measured reflectance value may correspond to two or more possible thickness values; this ambiguity can be avoided if a color camera is used.^12^ Third, calculated thickness from BAM depends on the lipid refractive index, which may need to be assumed. For HRCM, if layers of different thickness are present in an image, an estimate of lipid refractive index may be obtained,^12^ and so a more certain measure of thickness can be obtained. A third imaging method, atomic force microscopy (AFM),^13^ has better thickness and lateral resolution than an HRCM. However, an AFM provides only a single image at one time point and so cannot follow the dynamics of changes in meibum layer structure; therefore, HRCM was preferable for this study.

Studies of meibum spread on saline are often performed by dissolving the meibum in a solvent, such as chloroform, and then spreading the solution on saline, so that meibum is spread over the surface after the solvent has evaporated. However, because meibum contains stacked lamellae,^3^ these are likely to be disrupted by the solvent, so that the structure of the meibum spread on saline may be different from neat meibum spread on saline. This may explain why BAM images for neat meibum spread on saline^3^ show isolated patches of meibum within a dark surround, whereas BAM images derived from meibum dissolved in chloroform^14^^,^^15^ show a more continuous, network like structure (the above images were obtained below 30°C and at low surface pressures). In addition, it is notable that, after spreading meibum or a model lipid mixture dissolved in a solvent, the evaporation retardation of the film may increase for 24 hours or more,^16^ perhaps because the lipids are reorganizing into lamellar structures. For these reasons, neat, undiluted meibum, rather than a meibum solution, was spread on saline in the current studies.

The purpose of this pilot study was to study the predictions of the crystallite and evaporative models by imaging human meibum spread on saline as it cooled from eye temperature to below 30°C and to room temperature. The temperature range includes those near 30°C when the 11.1 nm lamellae at eye temperature would be expected to be replaced by 4.88 nm lamellae below 30°C.^3^ The following questions were considered:

1. Was there evidence for extensive stacked lamellae of either about 11.1 nm thickness above 30°C or of about 4.88 nm thickness below 30°C?
2. Do the results support either the “crystallite” model^5^ or the “evaporation” model?^7^

## Methods

The study adhered to the principles of the Declaration of Helsinki and was approved by the Biomedical Institutional Review Board of the Ohio State University. Informed consent was obtained from all subjects after explanation of the procedure. Subjects were eligible for the study if they were older than 18 years and had not worn contact lenses for 3 months before the study. Subjects were asked to avoid using cosmetic products on the day of the study. To provide a broad measure of the adult population, possible cases of dry eye were not excluded, but the study was not powered to show statistical differences between subjects with and without dry eye.

Ten subjects were enrolled, 7 women, age 29 ± 13 years (mean ± SD). Presence or absence of dry eye symptoms was assessed using the Ocular Surface Disease Index (OSDI) survey (Allergan, Inc., Irvine, CA, USA). Based on OSDI scores, one subject was classified as having dry eye.

### Procedure

**Figure 1.**
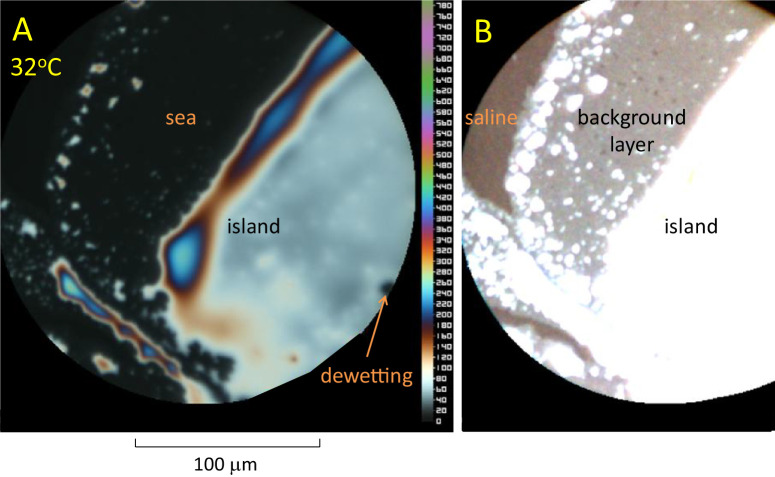
High resolution images of meibum spread on saline recorded at 32°C. The subject is a 62 year old White female with normal OSDI score. (**A**) Scale on the right indicates lipid thickness in nm, assuming a refractive index of meibum for the *d* spectral line of 1.477.^19^ (**B**). Same image with greatly increased contrast. See text for details.

Images were obtained with a high resolution, stroboscopic, color camera designed for in vivo images of the lipid layer; lateral resolution was about 0.5 µm.^11^ The original monochrome camera had been replaced by a color camera.^17^ The microscope was remounted so that the optical axis of the microscope objective was vertical. For each video recording of 100 images, surface temperature was recorded by an infrared thermometer to avoid contamination. The sharpest images in each recording were selected, based on the sharpness of the image surround edge,^11^ and used for further analysis. The contrast of images presented here have been increased by a factor of 1.8, except for Figure 1B, where the contrast was greatly increased to show the “background layer.”

A buffered saline was prepared as described by Mirejovsky et al.^18^ but without lipids or proteins, and used to fill a 50 mL PTFE beaker. The beaker was placed on a three-dimensional positioning stage to adjust for focus and image movement. Roughly, one sample of 0.015 µL of meibum was collected in the tip of a 0.5 µL glass capillary, using gentle pressure on the subject's lower lid. An initial recording was then made from bare saline at room temperature. After heating to about 40°C, a further recording from bare saline was made. The meibum was then pushed out on to the saline using a clean wire, and recordings of the spread meibum were made as soon as it could be located. The study was not intended as representative of what happens on the eye where the lid pull initiates and sustains blinking.

### Image Presentation and Analysis

Images presented here were chosen to illustrate the range of dynamics in meibum spreading, dewetting, and lamellar characteristics.

Spectral calibration of the imaging system is described in the Appendix. The derivation of responses of the three colors of pixels as a function of the thickness and refractive index of a layer of meibum on saline is also described in the Appendix. A method of using the observed responses of the red and blue pixels to estimate meibum thickness and refractive index is presented in the Results section. A complete exposition of the spectral calibration is given in the Appendix.

## Results

Images and results will be presented in order of decreasing temperature as the meibum cooled from near eye temperature and above about 30°C, to below 30°C and near room temperature.

### Images and Results Above About 30°C

Figure 1A is an example of how, initially, meibum spreads as a large “island” of irregular outline and thickness; the contrast has been increased to show details. A scale of lipid thickness in nm is given on the right; it is seen that the lipid thickness over most of the area is about 100 nm, but is bordered by a ridge of up to about 240 nm. Outside the island is a dark “sea” corresponding to thin or missing lipids. A small region of dewetting is seen at the lower right. Figure 1B shows the same image but with the contrast greatly increased to show details of the sea; it is seen that the sea consists of two components, a “background layer” of lipids next to the island and outside that, a layer considered to be saline or lipid that is too thin (e.g. about 1 nm or less) to be measured by the interference method. The background layer is seen to include numerous thicker inclusions, implying a nonuniform structure of meibum. The thickness of uniform areas of the background layer was estimated to be 7.82 ± 0.28 nm, (mean ± SD, *n* = 8 subjects) assuming its refractive index is 1.477.^19^ It should be noted that the estimated thickness depends inversely on the assumed value of refractive index – a higher value of refractive index would give a thinner background layer thickness.

**Figure 2.**
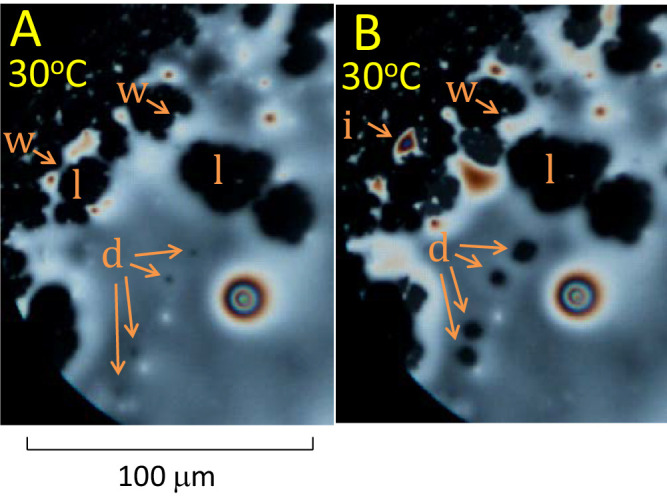
Dewetting of the lipid layer illustrated by images from 2 sequential video recordings, separated by an interval of 1 minute. The subject is a 21 year old White female with normal tear film. (**A**). **d** = “dots,” small holes in the meibum layer are the beginning of dewetting; **l** = “lakes,” larger, irregular holes form after expansion and fusion of dots; **w** = “walls” of lipid separate lakes from each other and the surrounding sea. (**B**) **d** = the dots have expanded, and moved closer together due to contraction of the lipid mass; **w** = walls have thinned and can break, so lakes can combine with each other or the surrounding sea; **i** = an “island” is formed after breaks and contraction of the surrounding walls.

Dewetting of the meibum layer is illustrated in Figure 2; the nomenclature of a previous paper on high resolution microscopy on the human TFLL will be used.^11^ Initially, small “dots,” dark holes in the meibum layer, are seen, such as the four dots labeled **d** in Figure 2A. These dots have expanded at the later time of Figure 2B; they are also closer together indicating general contraction of the lipid mass. In due course, some dots will fuse together to form larger, irregularly shaped “lakes,” **l**, Figures 2A and 2B. “Walls,” **w**, surrounding lakes, thin and break as the lakes expand, – compare Figure 2B with 2A. When a wall breaks, it may combine two lakes or connect a lake to the surrounding sea. Breaks in walls, can also generate small islands, **i**, Figure 2B.

**Figure 3.**
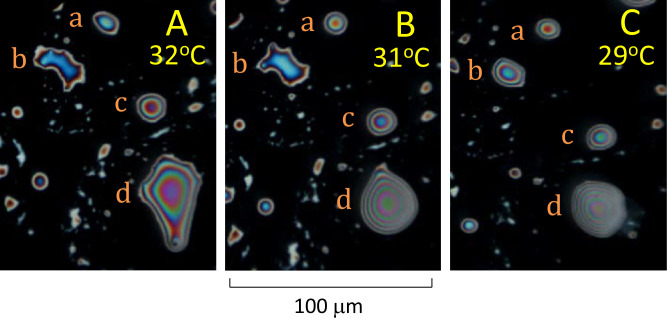
Later stages of dewetting illustrated by images from three video recordings. (**B**) and (**C**) were recorded 3 and 10 minutes after (**A**), respectively. The subject is a 27 year old White female with normal tear film. Panels (**a**, **b**, **c**, and **d**) are four islands which tend to develop circular outlines, becoming “lenses” at later times. See text for details.

**Figure 4.**
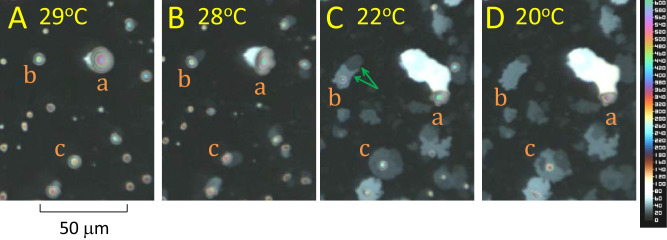
Formation and development of “tails” below about 30°C. The subject is a 27 year old White male with normal tear film. The four panels, (**A** to **D**), show changes in three lenses, panels (**a**, **b**, and **c**), at four different times and temperatures, as indicated. Scale of lipid thickness in nm is on the right. See text for details.

**Figure 5.**
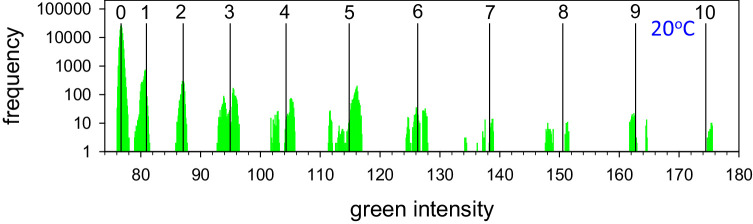
Histogram of averaged intensities of green pixels in uniform 5 × 5 pixel areas. The subject is a 27 year old White male with normal tear film – the same subject as in Figure 4. Histogram is derived from images from eight video recordings. Vertical lines indicate expected green intensities for 0 up to 10 lamellae of 4.73 nm thickness, as indicated, based on spectral calibration of the optical system – see the Appendix. Note the logarithmic scale of frequency. See text for further details.

**Figure 6.**
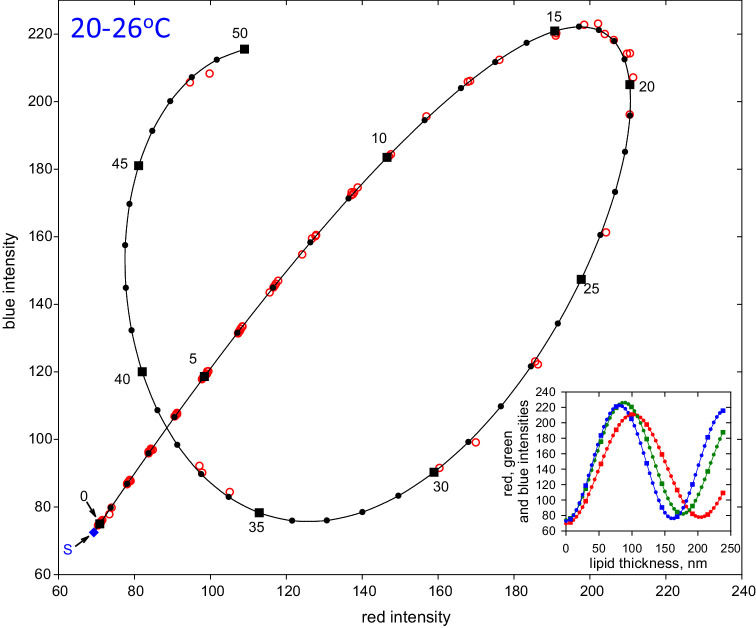
Red circles show averaged blue intensities as a function of averaged red intensities in uniform 5 × 5 pixel areas (sum of red, green, and blue variances less than a specified criterion); the inset provides an explanation of the derivation of the main plot. These circles correspond to means from 13 video recordings. The subject is a 62 year old White female with normal tear film – the same subject as in Figure 1. *Black dots* on the *black curve* show predictions for lipid layers containing multiple lamellae of 4.77 nm thickness superimposed on a background layer of 6.8 nm thickness. Refractive index of the lipid layers was assumed to be 1.497 for the **d** spectral line with a constringence of 50 based on the **C** and **F** spectral lines.^23^ *Black squares* and *numbers* indicate multiples of five layers and corresponding squares and dots have been added to the inset. Blue diamond labeled **S** gives predicted red and blue intensities for bare saline (zero lipid thickness in the inset) which was not seen in these images.

A later stage of dewetting is illustrated in Figure 3 – the 3 images are taken from recordings at different times. At the earliest time, panel A, islands **b** and **d** have irregular outlines and contours, as in the island in Figure 2B. At the later time in panel B, island **d** is more nearly circular and lens shaped, whereas island **b** remains quite irregular. By the time of panel C, island **b** has also become nearly circular and lens shaped. Island **a** is already fairly circular in panel A, and becomes more circular in panel B. Island **c** is already quite circular in panel A. Thus, islands tend to be irregular when they first form but become circular lenses at later times. It should be noted that these lenses do not show the characteristics of tightly stacked lamellae described later in Figures 4, 5, and 6. All four islands become thicker centrally, due to contraction of the islands. The fact that the formation of islands occurs at different times indicates some variability in meibum composition between islands. The results of Figures 2 and 3 were chosen because they illustrate the various stages of dewetting; not all subjects showed all these stages of dewetting, but meibum from all 10 subjects formed such lenses and hence showed lipid dewetting. Many of the above observations about dewetting have been reported in a study of duplex (i.e. relatively thick) films of oils.^20^

### Formation of “Tails” Below About 30°C

Below 30°C, lenses start to grow “tails” with a multilamellar structure. Figure 4 shows typical development of these tails over 4 times and temperatures. In Figure 4A, three lenses are labeled, lenses **b** and **c** having a circular outline, whereas lens **a** shows the beginning of secretion of a thick (bright) tail on the left side. By Figure 4B, this thick tail of lens **a** has expanded toward the left. The right side of lens **a** has become distorted, as if parts of it are being pulled by the tail via internal connections. Lenses **b** and **c** and some other lenses have started to grow thin (dim) tails. Lenses appear to be pushed along by the tails – note how the distance between lenses **a** and **b** have increased, whereas the distance between the tips of the tails tends to remain constant – see the tails of lenses **a** and **b** in Figures 4B, 4C, and 4D. It seems that the tails bond to the underlying background layer, while the lenses can move along over the background layer. In Figure 4C, the thick tail from lens **a** has considerably elongated and has developed a kink as the new lipid is secreted in a more vertical direction. Lens **b** is adding a second layer on top of the original tail (green arrows), neatly following the original outline. The tail of lens **c** now surrounds the lens like a halo. By Figure 4D, lens **b** has been converted so all the material from this lens in Figure 4A has now been incorporated into the tail with perhaps some also added to the background layer.

Figure 4 demonstrates considerable differences between the structure of lenses and tails. Lenses have a rounded convex structure indicating (nearly) isotropic fluid properties.^21^ By contrast, tails often have a flat surface and sometimes show signs of layers stacked on each other – see the tail of lens **b** in Figure 4C (green arrows). To test whether the tails are composed of stacks of lamellae, Figure 5 illustrates a histogram of green intensities derived for small, 5 × 5 pixel, uniform areas (sum of red, green, and blue variances less than a specified criterion, thus avoiding pixels near edges of tails). Green pixels were used rather than red or blue, because there were more green pixels than the others, but similar plots were obtained for red and blue – not shown. It is seen that green intensity in the tails was “quantized,” that is, the histogram consists of a number of peaks, rather than a continuous distribution. For lipid thickness of less than a quarter wavelength, as indicated by the color of the lipids used in Figure 5, intensity is an increasing function of lipid thickness^11^ – see inset to Figure 6; thus the quantization of intensity indicates a corresponding quantization of lipid thickness, as expected from a stack of lamellae. Vertical bars, labeled 0 to 10, in Figure 5 correspond to predicted intensities for stacks of 0 up to 10 lamellae of 4.73 nm thickness. The spectral calibration of the optical system is described in the Appendix (further details of the assumptions made in Figure 5 are given in Figure 6 and its discussion). It is seen that the histogram clusters tend to match the predicted intensities based on stacks of lamellae. The cluster corresponding to three lamellae has two peaks and the clusters corresponding to four to nine lamellae show separated subclusters; this indicates that the lamellae may have somewhat variable thickness. The subclusters are not due to variability in illumination, because the standard deviation of intensity for zero lamellae is only 0.21 units, indicating good illumination uniformity – the separation of some of the subclusters can be seen to be much greater than this.

The tails for the subject in Figure 5 were relatively thin – generally less than a quarter wavelength thick and so were colored grey or very pale blue – see Figure 4. For other subjects, the tails were sometimes greater than a quarter wavelength thick; because intensity is an oscillatory function of lipid thickness,^11^ green intensities could correspond to different thickness values and so histograms of green intensities became difficult to interpret. A better method of analyzing thick tails is to plot blue intensities on the vertical axis and red intensities on the horizontal axis, as shown by the red circles in Figure 6. This figure can be understood by noting the difference between the responses of red and blue pixels as a function of meibum thickness, shown in the inset to Figure 6; both functions are an oscillatory function of thickness, with peaks at a quarter wavelength thickness and minima at zero and half wave thickness, etc.,^22^ but blue pixels respond to shorter wavelengths than red pixels so the two oscillations become more out of phase as the thickness increases. This method has been described in the supplemental material of Reference 12 and a more complete exposition is given in the Appendix. As in Figure 5, the circles were derived from averages for 5 × 5 uniform areas; each circle corresponds to means of groups of such averages from 13 video recordings from one subject. Black dots on the black curve correspond to predicted intensities assuming the tails are composed of stacks of lamellae of 4.77 nm thickness superimposed on a background layer of 6.8 nm thickness. Black squares and numbers indicate multiples of five layers. The blue diamond labeled **S** corresponds to estimated intensities for bare saline, which was not observed in these images. It is seen that the observed data, red circles, are in generally good agreement with the predicted values, black symbols, so that the number of layers for each observation can be determined, except near the upper right end of the black curve where the predicted values are close together (about 17 or 18 lamellae). An advantage of this method is that the fitted curve involves estimation of the refractive index and dispersion of the tails, thus reducing the uncertainty in estimated lipid thickness due to unknown refractive index; the refractive index for the **d** spectral line was estimated to be 1.497, rather greater than the value of 1.477 for meibum,^19^ and dispersion, constringence,^23^ based on **C** and **F** spectral lines was estimated to be 50. Average lamellar thickness was 4.82 ± 0.13 nm (*n* = 8) and was not significantly different from 4.88 nm, the thickness of the SPP found by small angle x-ray scattering.^3^

## Discussion

**Figure 7.**
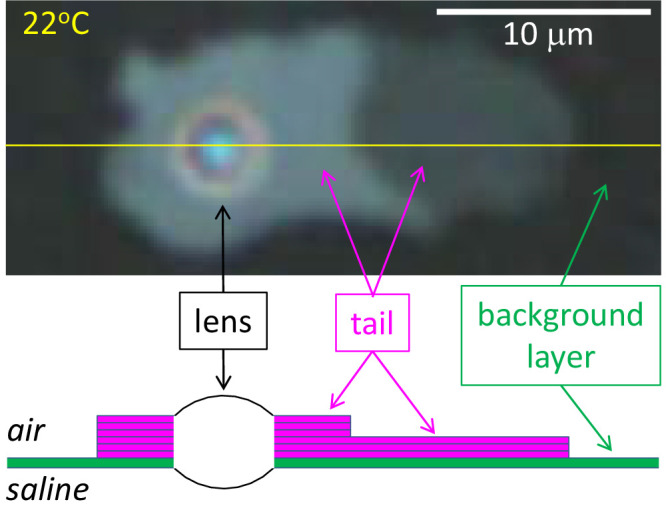
Interpretation of a section of the tails from lens **b** in Figure 4C (vertical scale is exaggerated compared to horizontal scale and the tail lamellae should be only about 2% of the central thickness of the lens – about 4.8 nm compared to about 240 nm). See text for discussion.

Some major findings of this study are, first, Figure 1 shows that the initial irregular island emits a thin background layer of about 7.8 nm thickness (value depends on the assumed value of refractive index). Second, Figures 2 and 3 show that a process of lipid dewetting follows a pattern seen in some (but not all) in vivo images^11^ leading eventually to lipid “lenses”; similar dewetting has been described in duplex films.^20^ Third, Figures 4, 5, and 6 show that when the lipid has cooled to below about 30°C, these lenses start to emit tails that are stacks of up to 49 lamellae of 4.82 ± 0.13 nm thickness each, which is not significantly different from the thickness of SPP lamellae found by small angle x-ray scattering.^3^ These findings are summarized schematically in Figure 7 (not drawn to scale), which illustrates a section through lens **b** in Figure 4C (after rotation). Initially, three lamellae extended on the background layer toward the right (see Fig. 4B) but later a further three lamellae spread over the original lamellae. Eventually, as shown in Figure 4D, the lens disappears, so all the meibum has been incorporated either into the background layer or the tails. For these experiments, this indicates that below 30°C, meibum can be considered to have two components – the background layer and tails.

The thickness of the lamellae in tails is not significantly different from the thickness of the SPP lamellae, 4.88 nm, found by x-ray scattering.^3^ Thus, in answer to question 1 of the beginning of this paper, below 30°C, we found evidence of extensive stacked lamellae, with a thickness similar to the SPP from x-ray studies.^3^ Therefore, our method is capable of demonstrating stacked lamellae of this thickness. However, above 30°C, we found no evidence for tightly stacked lamellae similar to the 11 nm thickness of the LPP of x-ray studies.^3^ This is consistent with in vivo images for which, similarly, stacked lamellae were not evident.^11^ It is probable that lamellae of this thickness were present because they were evident in the x-ray studies^3^ and lamellae of rather similar thickness were demonstrated by electron microscopy in mouse meibum.^1^ An explanation of the absence of stacked lamellae in the meibum film above 30°C is that lamellae may be separated by a more fluid, disordered meibum of variable thickness.

These findings may be considered in combination with the results of a previous study which measured thinning rates of the tear film as a function of lipid thickness.^24^ The thinning rate of the tear film is an estimate of the evaporation rate.^25^ Local thinning rates were found to have a bimodal distribution with peaks at 1.6 and 8.4 µm/min, indicating a corresponding bimodal distribution of evaporation rates.^24^ Gaussian curves fitted to the 2 peaks intersected at a thinning rate of 4 µm/min, so thinning rates above and below this value were described as “rapid” and “slow,” respectively. It was found that all 5 eyes with TFLL thickness less than 24 nm showed rapid thinning whereas 31 of 33 eyes with lipid thickness greater than 30.5 nm showed slow thinning.^24^ It may be noted that the effect of lipid thickness on evaporation is quite strong^24^; the 5 eyes with lipid thickness in the range 16 to 24 nm gave a mean thinning rate of 8.2 ± 2.0 µm/min, whereas the mean thinning rate for twice this thickness, 21 eyes in the range 32 to 48 nm was only 1.83 ± 0.93 µm/min, a factor of 4.5 times lower. According to a model of diffusion of water molecules through a disordered lipid film,^20^ this factor would be expected to be less than 2.0, indicating that the TFLL involves more than disordered lipids.

**Figure 8.**
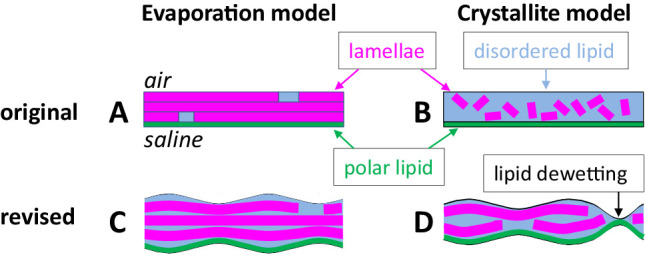
Proposed revisions to the evaporation and crystallite models. See text for details. (**A**) Original evaporation model. (**B**) Original crystallite model. (**C**) Revised evaporation model. (**D**) Revised crystallite model.

Figure 8 shows revised models of the TFLL based on the results of the current study and the relation of rapid and slow evaporation to TFLL thickness. Our original “evaporation” model, Figure 8A, contained tightly stacked lamellae^7^ as in the skin lipid layer.^4^ Figure 8B represents the “crystallite” model of randomly oriented, tiny crystals of lamellar lipid within a disordered visco-elastic continuous phase.^3^ Figures 8C and 8D show revisions of both these models that attempt to match the characteristics of “slow” and “rapid” evaporation, respectively. In the revised models, the evaporation model, Figure 8A, has been modified, see Figure 8C, to include variable thickness of disordered lipid between the lamellae, explaining why tightly stacked lamellae were not observed in this study above 30°C or in in vivo studies.^17^ The crystallite model, Figure 8B, was revised, see Figure 8D, by including information that any patches of lipid lamellae are much greater in extent than the thickness of the TFLL^1^^,^^26^ indicating that the lamellae must lie parallel to the surface rather than being randomly oriented.

Figure 8C is a model of the TFLL for a thickness greater than 30.5 nm, and hence tending to yield slow thinning and evaporation rates,^24^ as noted above. The thickness of the LPP (eye temperature) lamellae is 11.1 nm so TFLL of this thickness range may contain an average of at least two lamellae in addition to the polar lipid layer and the disordered lipid between lamellae. Figure 8D is a model of the TFLL for a thickness of less than 24 nm – in this case, the average number of lamellae is probably less than 2 given consideration of the 11.1 nm lamellar thickness and the contribution of the polar lipid layer and disordered lipid. Even a single lamella might be expected to be a moderate barrier to evaporation, that is, half of the evaporation resistance of two lamellae, so it is suggested that the high evaporation rate found in these thin TFLLs^24^ may be caused by dewetting, as illustrated in Figure 8D. It should be noted that dewetting of layers is caused by van der Waals’ forces between the exterior and interior surfaces of the layer^27^ and these forces increase rapidly with reduction in layer thickness; the van der Waals’ force between two surfaces is inversely proportional to the cube of the distance between the surfaces.^28^ Perhaps when at least 2 lamellae are present, see Figure 8C, these forces are not strong enough to disrupt the lamellae, whereas when only a single lamella is present in some regions, see Figure 8D, these forces are strong enough to disrupt the lamella and cause dewetting.

These results suggest that a more extensive study including a comparison of patients with dry eye with healthy subjects is warranted. A limitation of our study is that the effects of time and temperature are confounded – it is not clear how observed changes are related to time rather than temperature. A further limitation is that the study does not include polar lipids that are contained in the aqueous layer and help to form the interface between nonpolar lipids and the aqueous layer. Future high-resolution imaging of meibum on saline should include the ability to control temperature with both cooling and heating and could also control surface pressure in a Langmuir trough. It would also be interesting to use transmission in addition to reflection microscopy, for example, to use polarization methods to study meibum birefringence.^26^ Additionally, a comparison of in vivo and in vitro imaging could provide important insights into mechanisms of evaporative dry eye. A better understanding of the structure of the TFLL is surely important in providing insight into the origin of evaporative dry eye.

## Figures and Tables

**Figure A1.**
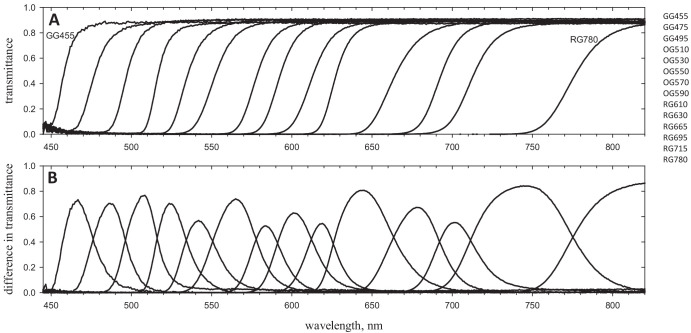
(**A**) Transmission spectra of long pass filters used for spectral calibration of the optical system. The filter used are listed on the right. (**B**) The transmission spectra of “virtual filters” derived from differences between transmittance of long pass filters in panel **A**. See text for discussion.

**Figure A2.**
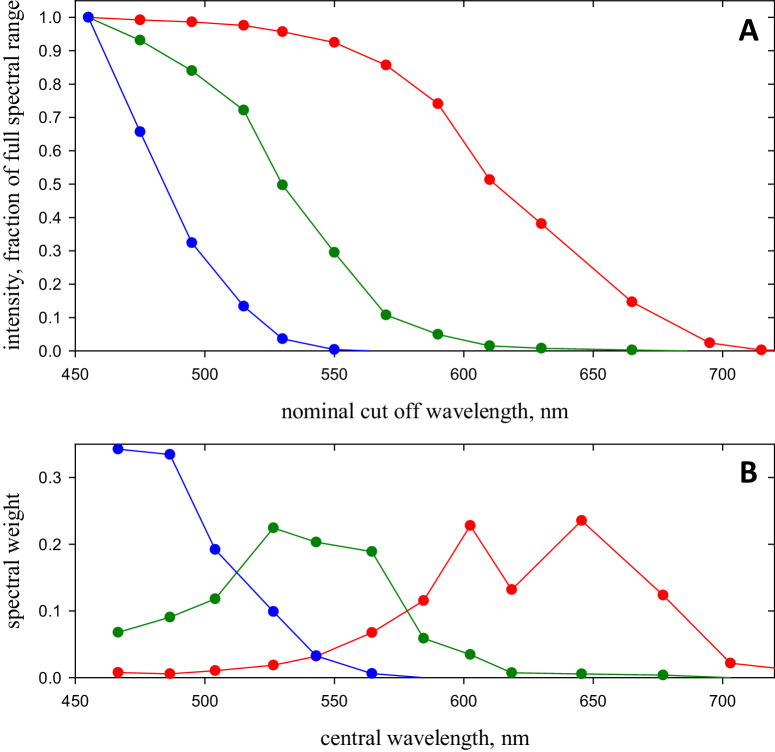
(**A**) Responses to reflection from an uncoated BK7 glass surface, of the red, green, and blue pixels to the long pass filters in Figure A1A. (**B**) Spectral weights of the virtual filters of Figure A1B, derived from differences between responses from the long pass filters in panel **A**. A small correction for the variation in BK7 reflectance with wavelength has been made. See text for discussion.

## Appendix Appendix: Calibration of the spectral weighting functions of the imaging system

Here, we describe in detail how to calibrate the optical system to determine the thickness, and thus any layering of meibum that may be present in the experimental images. This appendix combines results from previous studies,^22^^,^^24^ and adapts the method to meibum on saline.

To derive meibum thickness from measurements of green, red, and blue intensities, as in Figures 5 and 6, requires knowledge of the spectral weighting functions of the imaging system for the 3 colors of pixels, that is, the overall sensitivity of the system as a function of wavelength. The spectral weighting function, *w*(λ), for any of the three colors of pixel depends on the product of (i) the power spectrum of the stroboscopic light source, (ii) the transmittance and reflectance spectra of components in the illumination and imaging optical paths, and (iii) the spectral sensitivity of the camera sensor for that color of pixel. The response, *V*, of any color of pixel to reflection from a surface of reflectance, *R*(λ) is given by

(A1) $$\begin{array}{c} V=k\int_{\lambda_{1}}^{\lambda_{2}}w\;\left(\lambda \right)R\;\left(\lambda \right)d\lambda \; \end{array}$$

where the constant *k* can be determined by measuring response from a surface of known spectral reflectance, such as a clean, uncoated glass surface.^22^

A possible method for estimating the spectral weighting functions would be to measure the camera responses to reflection from a surface of known reflectance spectrum for a series of narrow band interference filters of known transmission inserted into the illumination beam, and so estimate the spectral weighting functions by dividing the measured responses by the integrated transmission of the filter. Thus, from Equation A1,

(A2) $$\begin{array}{c} V^{'}=k\int_{\lambda_{1}}^{\lambda_{2}}w\;\left(\lambda \right)R\;\left(\lambda \right)t\;\left(\lambda \right)d\lambda \; \end{array}$$

where V' is the response of the color pixel and t(λ) is the transmittance spectrum (λ_1_ <  λ  <  λ_2_) of the filter. If it is assumed that *w*(λ) and *r*(λ) are constant or are slowly and smoothly varying functions of λ, then

(A3) $$\begin{array}{c} V^{'}≅kw\;\left(\lambda^{'}\right)R\;\left(\lambda^{'}\right)\int_{\lambda_{1}}^{\lambda_{2}}t\;\left(\lambda \right)d\lambda \; \end{array}$$

where λ*′* is the central wavelength of the interference filter. Thus, an estimate of the weighting function, w′(λ′), is given by

(A4) $$\begin{array}{c} w^{'}\;\left(\lambda^{'}\right)=\frac{V^{'}}{kR\;\left(\lambda^{'}\right)\int_{\lambda_{1}}^{\lambda_{2}}t\;\left(\lambda \right)d\lambda }\; \end{array}$$

These estimated weighting functions could now be interpolated between the central wavelengths, λ', to give an estimated weighting function, *w*(λ), and used to calculate the relation between response *V* and reflectance *R*(λ) using Equation A1.

The power spectrum of the stroboscopic light source has a number of large, narrow peaks, thus tending to invalidate the assumption in Equation A3 that the weighting function, *w*(λ), is a slowly and smoothly varying functions of λ. For example, if there is a gap in the coverage of the spectrum between the pass bands of two interference filters and there are more or larger peaks in the gap than within the pass bands of the two filters, the weighting function in this region of the spectrum will be underestimated.

To avoid this problem of spectral gaps, the following strategy was used. The imaging system includes a Schott GG455 long pass filter to reduce the effect of chromatic aberrations at short wavelengths. The responses of the three colors of pixels, to reflection from a glass surface, was measured first with this filter in place, and then after replacing it consecutively with a series of 13 long pass filters listed at the upper right of Figure A1. The transmittance spectra *t*_*i*_(λ) of these filters are shown in Figure A1A. Figure A1B shows the differences between transmittance spectra between neighboring spectra, for example, the leftmost curve show the transmittance spectrum of GG455 minus that of GG475, $t_{1}\left(\lambda \right)-t_{2}\left(\lambda \right)$; (the rightmost curve in Figure A1B shows the transmittance spectrum of RG780, but this was not relevant to the analysis because there was no detectable response of the three colors of pixels with this filter).

The peaks in Figure A1B can be considered “virtual filters” whose summed transmittance spectra equals that of GG455; therefore, these virtual filters do not suffer from the effects of gaps in the spectral coverage that could be a problem when using interference filters. The responses of the 3 colors of pixels to these virtual filters can be determined by first measuring their responses, *V_i_, i* = 1,... 14, to the series of long pass filters to reflection from an uncoated BK7 glass surface of specified refractive index and dispersion – these responses (as a fraction of the response of the system with the GG455 filter) are shown in Figure A2A. Each dot may be considered to come from evaluating equation A1. The “virtual response” to the virtual filters, *U_i_*, is then given by the difference between the responses to neighboring long pass filters; thus, for the first pair of filters,

(A5) $$\begin{array}{c} U_{1}\;=\;V_{1}\;-\;V_{2}.\; \end{array}$$

Based on Equation A1, this virtual response can be considered to be proportional to the product of the spectral weight, *w_i_*′ of the filter times the reflectance, *R_i_*, of the BK7 glass surface at the central wavelength of the virtual filter. Therefore, the spectral weight for a virtual filter is given by

(A6) $$\begin{array}{c} w_{i}=\frac{KU_{i}}{R_{i}}.\; \end{array}$$

Where *K* is a constant. The spectral weights from Equation A6 have been plotted in Figure A2B where *K* has been scaled so that the sum of the weights is 1.0 for each color of pixel using BK7 glass; the factor is

(A7) $$\begin{array}{c} K=\frac{1}{\sum_{i=1}^{13}\left(\frac{U_{i}}{R_{i}}\right)}.\; \end{array}$$

To calculate the expected responses of each of the three colors of pixels to a certain thickness of meibum, the integral of Equation A1 was replaced by a summation over all the virtual filters of the form

(A8) $$\begin{array}{c} U=\sum_{i=1}^{13}w_{i}R_{mi}\;\left(h,{\lambda_{i}}^{'}\right)\; \end{array}$$

where *w_i_* is the spectral weight given in Figure A2B and *R_mi_*(*h*, λ_*i*_′) is the predicted reflectance for that meibum thickness *h* and central wavelength λ_*i*_′ of the virtual filter *i*. The predicted reflectance for a given meibum thickness and wavelength can be derived from Equations A2, A7, and A11 of a previous publication.^22^

Using those equations, the reflectance of the meibum layer (a high index layer^1^) may be computed from

(A9) $$\begin{array}{c} R_{mi}=R_{m,av}\left[1+C\cos\left(\left(4\pi n_{m}h/{\lambda_{i}}^{'}\right)+\pi \right)\right],\; \end{array}$$

where

(A10) $$\begin{array}{c} C=\frac{2\alpha }{1+\alpha^{2}},\; \end{array}$$

and

(A11) $$\begin{array}{c} \alpha =\left|\frac{r_{2}}{r_{1}}\right|,\;r_{1}=\frac{n_{a}-n_{m}}{n_{a}+n_{m}},\;\text{and}\;r_{2}=\frac{n_{m}-n_{s}}{n_{m}+n_{s}}. \end{array}$$

Here *n_a_* = 1.0 is the index of refraction of air, *n_m_* is the index of refraction of meibum, *n_s_* = 1.337 (corrected for the optical dispersion of saline for the red and blue pixels) is the index of refraction of saline, and *r*_1_ and *r*_2_ are the amplitude reflection coefficients of the air and saline surfaces of the meibum layer. The value for *n_m_*, one value for each of blue and red, resulted from optimizing the fit of the theory to experiment for the red and blue pixels in Figure 6. The optimum value for red is *n_m_* = 1.496, and for blue we found *n_m_* = 1.504. We also have

(A12) $$\begin{array}{c} R_{m,av}=r_{1}^{2}+r_{2}^{2}.\; \end{array}$$

Finally, the optimization included finding the optimum values of the response from bare saline for red and blue pixels, shown by the diamond in Figure 6.

The close fit of the observed blue and red intensities to the predicted curve in Figure 6 indicates the accuracy of this spectral analysis of the imaging system.
